# Antibiotic consumption in 14 countries of sub-Saharan Africa: Findings from a retrospective analysis

**DOI:** 10.1371/journal.pone.0333842

**Published:** 2025-10-30

**Authors:** Yvon de Jong, Rachit Singhal, Yewande Alimi, Geetanjali Kapoor, Abdourahmane Sow, Martin Matu, Edwin Shumba, Manuel Moreira, Deepak Batra, Ramanan Laxminarayan, Pascale Ondoa, for the MAAP study group

**Affiliations:** 1 IQVIA, Centurion, South Africa and Dubai, UAE; 2 Africa Centers for Disease Control and Prevention (Africa CDC), Surveillance and Disease Intelligence Division, African Union, Addis Ababa, Ethiopia; 3 One Health Trust, Washington, DC, WA, United States of America,; 4 West Africa Health Organization, Bobo Dioulasso, Burkina Faso; 5 Eastern, Central and Southern African Health Community, Lusaka, Zambia; 6 African Society for laboratory Medicine, Addis Ababa, Ethiopia; 7 Innovative Support to Emergencies, Diseases and Disasters (InSTEDD), Seattle, Washington, United States of America; 8 Amsterdam Institute for Global Health and Development, Department of Global Health, University of Amsterdam, Amsterdam, The Netherlands; University of Business and Technology, ALBANIA

## Abstract

**Background:**

Antimicrobial consumption (AMC) measures the level and types of antibiotics consumed in a specific setting. Monitoring AMC is critical component of antimicrobial resistance (AMR) containment strategies. However, AMC data at both facility and national-levels are scarce in Africa, which limits our understanding of the rate, patterns and drivers of antibiotic consumption, and prevents the establishment of evidence-based antimicrobial stewardship.

**Methods:**

We determined facility and national-level rates and patterns of AMC from data retrospectively collected between 2016 and 2019 in 327 pharmacies from 14 countries AMC data collection followed a backfilling strategy leveraging from public and private central medical stores, wholesalers, distributors or import services as data sources. Participating hospital and community pharmacies were selected based on their location in or proximity to hospitals capable of producing AMR data. Levels of AMC were determined as defined daily dose (DDD) and DDD per inhabitant (DID). AMC patterns were analysed according to the WHO Access, Watch, and Reserve (AWaRe) Categories, the Anatomical Therapeutic Chemical (ATC) classes and the individual antibiotic molecules included in the Drug Utilisation 75% (DU75). The availability of antibiotics was examined against the WHO and the National Essential Medicine Lists (EML).

**Results:**

National AMC data was available in 11 of the 14 participating countries, revealing a collective AMC of 8.42 DID varying from 2.8 to 115.5 at individual country level. AMC was also determined in 327 hospital and community pharmacies. Nine of 11 (82%) countries with national data available, and 219 of the 327 (72%) participating pharmacies achieved the WHO AWaRe target of at least 60% of antibiotic consumption from Access drugs. Eighty percent of country-level AMC was accounted for by five ATC sub-classes classes of antibacterial for systemic use. Facility-level antibiotic consumption was dominated by a narrow scope of less than five drugs, taking advantage of only 10% of all possible WHO-recommended Access drugs within ATC classes. Collectively, the 14 national EML included 70% of Access, 60% of Watch and less than 5% of Reserve antibiotics listed in the WHO EML. Forty-eight uncategorized and 50 categorized non-WHO-recommended drugs were included in national EMLs or documented to be circulating in countries.

**Interpretation:**

The relatively low AMC and the poorly diversified subset of antibiotics available in countries underscores that strategies to expand equitable access to adequate treatment of bacterial infections should complement current efforts to promote the judicious use of antimicrobials. Interventions to increase the volume of analysable data on AMU, AMC and AMR, should be prioritized in national AMR action plans as well as in wider infrastructural and economic development plans.

## Introduction

Antibiotics are essential medicines that are used to prevent or treat bacterial infections and improve morbidity and mortality [1] However, misuse and overuse of antibiotics are also identified as key drivers of antimicrobial resistance (AMR), which is among the top ten global public health threats faced by humanity [2]. The burden of AMR is also particularly high in LMIC of sub-Saharan Africa, with AMR-associated mortality rate up to 27.3 deaths per 100,000 [3].

Antibiotic consumption is demonstrated to be rising in LIMC but remains lower than in high income countries (HIC), despite higher burden of bacterial infections [3], posing the question of excess versus lack of access to antibiotics. Country and facility-level information on antimicrobial consumption (AMC) is critical to inform the design and monitor the effectiveness of AMR mitigation policies, including balancing interventions to reduce the injudicious use of antibiotics [4,5] while expanding equitable access to adequate treatment of bacterial infections.

The World Health Organization (WHO) recommends AMC surveillance as a key component of the Global Antimicrobial Surveillance system (GLASS) [4]. However, obtaining a complete overview of country-level AMC is a challenge in most LMIC of sub-Saharan Africa due to the limited capacity of their health systems and various technological barriers complicating routine data collection and analysis. Sources of information are usually scattered throughout the pharmaceutical supply chain, which includes importers, local manufacturers, distributers, wholesalers and community and hospital pharmacies.

Notwithstanding the fragmented national surveillance systems, understanding antibiotic consumption is crucial for assessing the relationship between antibiotic use and resistance over time locally, nationally and regionally [4]. Intelligence on AMC can inform stewardship strategies supporting the reduction of injudicious antibiotics use while ensuring equitable access to adequate treatment of bacterial infections. We postulated that valuable information on AMC can be obtained from existing in-country data sources and can inform in-country and regional AMR containment strategies, including interventions to strengthen AMC surveillance systems.

In this study, we report rates, trends and patterns AMC using data collected in the frame of the Mapping AMR and AMU Partnership (MAAP). MAAP is a consortium launched in 2018, with the aim to increase the volume of analyzable AMR and AMC datasets by retrieving and analyzing retrospective human health data from 14 African countries [6].

Based on their availability, accessibility and usability, retrospective AMC data were collected at national and facility level, and across the public and the private sector in the 14 participating countries. AMC patterns were analysed according to the WHO Access, Watch, and Reserve (AWaRe) Categories [7], the Anatomical Therapeutic Chemical (ATC) classes [8] as well as the individual antibiotic molecules contributing to the Drug Utilization 75% index (DU75). The availability of antibiotic was examined against the WHO and the National Essential Medicine Lists (EML) [9,10]. Here we discuss the implications of AMC data availability on the feasibility of AMC surveillance. We also examine the significance of AMC levels and profiles on antibiotics access and judicious use.

## Methods

### Study design

This was an analysis of retrospective data on antimicrobial consumption between 2016 and 2019 and conducted in the frame of the MAAP study described elsewhere [11,12]. Retrospective data collection took place between February 2019 and November 2020, at central and facility levels and across the public and the private sectors of 14 African countries prioritized by the Fleming Fund (S1 File supporting information) and participating in MAAP (Burkina Faso, Cameroon, eSwatini, Gabon, Ghana, Kenya, Malawi, Nigeria, Senegal, Sierra Leone, Tanzania, Uganda, Zambia and Zimbabwe).

### AMC data sources

**Fig 1:**
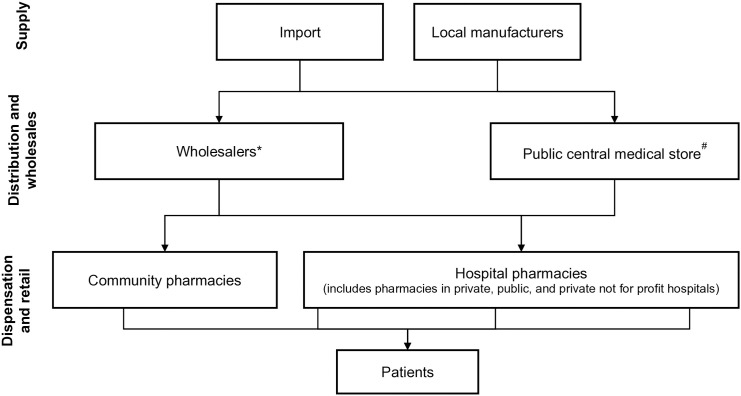
Flow of antimicrobial resistance through the health care system. *Includes distributors as well; # includes CMS: Central Medical Store; CPS – Central Purchasing Store; and NPS – National Pharmacy Supply.

**Fig 2:**
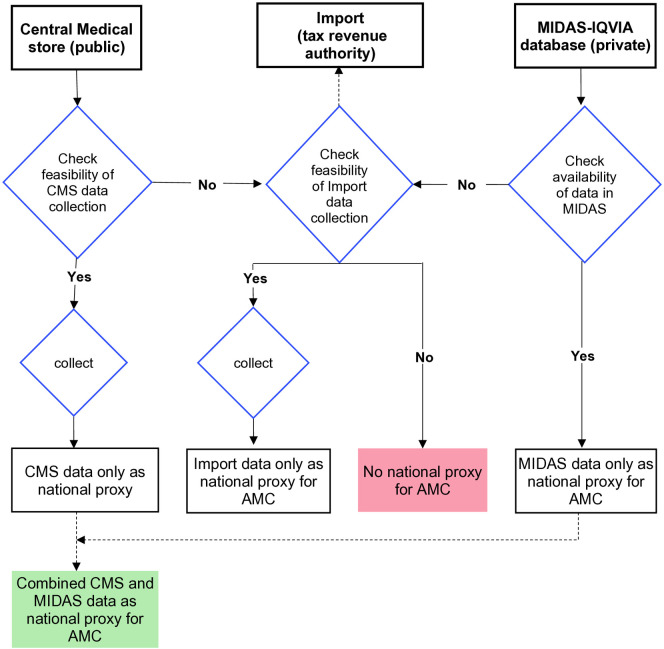
Backfilling strategy for the AMC data collection at national level. Data from central medical stores (CMS, public) were collected in first intention, whenever feasible. Pre-existing MIDAS-IQVIA data were included in the analysis. Data from the tax revenue authorities (import) were included only if no data from central medical stores or from MIDAS-IQVIA were available. The source of national AMC data represented one of the following (i) CMS only; (ii) MIDAS-IQVIA only; (iii) import only; (iv) combined CMS & MIDS-IQVIA; or (v) not available.

Schematically, antimicrobials flow through the healthcare system from import to distribution, to dispensation channels (**Fig 1**). The scope of AMC data collection included the distribution of antimicrobials at central level and the dispensation at facility level in public and private sector. Units responsible for import, purchase and distribution of antimicrobials in the public and private sectors were identified in collaboration with respective country authorities to comprehensively map the sources for national AMC data. National data on AMC were collected using a backfilling strategy [**Fig 2**]. The entry point for data collection were the Central medical store (public sector), whenever feasible, and the pre-existing data sets from the MIDAS database of IQVIA, when available. MIDAS tracks products in 693 therapeutic classes across 77 countries globally [13]. The coverage of MIDAS data is calculated by dividing the pharmaceutical market value of the audited data with the overall private sector market estimate collected from ad-hoc surveys and literature analysis conducted by IQVIA data production and statistics team. Import data were collected when feasible, in case no data from central medical stores could be collected and no data from wholesalers where available in the MIDAS database.

Facilities providing data at dispensation levels were the pharmacies located in the hospitals were MAAP AST data collection was conducted [11,12], as well as community (i.e., private) pharmacies located within a 5 km radius from the hospitals. Briefly, the selection of participating hospitals in the MAAP study was done as follows: the national AMR coordinating committees (AMRCCs) and laboratory governance officials provided the list of all hospital-based laboratories with reported bacteriology testing capacity in the 14 participating countries. The AMR detection capacity of each laboratory was evaluated through a self-administered electronic survey tool. The result of this survey was used to assign AMR detection readiness scores to laboratories. The final selection of laboratories was made in consultation with the respective AMRCCs, considering the laboratories’ AMR detection readiness scores, geographical coverage, and representativeness [12]. For budgetary reason, a maximum of 30 pharmacies per country was included in the analysis. Community pharmacies closest to the participating hospitals were pre-selected in priority. In case pre-selected pharmacies did not agree to share data or did not have a suitable system to collect AMC information, further community pharmacies located within the 5 km radius of the participating hospitals were approached for participation. Import units, private distributors and wholesalers, central medical stores and pharmacies were asked to confirm their willingness and capacity to provide retrospective data on AMC. The feasibility of data collection was assessed by the MAAP study team among participating units consenting to participate, based on the availability, accessibility and usability.

### Data collection

The core list of antimicrobials studied included J01 (antibiotics for systemic use) and P01AB (nitroimidazole derivates) and optionally selected J02 (antimycotics for systemic use) of the ATC Classification system [8].

Data from paper-based stock cards, bin cards or invoices or from the electronic information systems, were all collected and stored through electronic tools designed by inSTEDD to facilitate the digitization, storage, secured sharing and aggregation of data from various sources and formats (S2 File supporting information). Product details (such as molecule name and strength) of registered antimicrobials in scope in the country were pre-entered in the tool to standardize data collection. Data collection was conducted by local teams of 3–18 collectors, who had previously received standardized training in the use of the tools.

Data checks were performed on 100% of records by the in-country supervisor and the regional coordinator to ensure inclusion of key parameters, stock accounting for selected facility, and temporal checks (2016–2019). Queries were resolved by the data collectors, after which the central data team validated and standardized the datasets.

### Other data and document sources

Most recent national EML were collected from websites of Ministries of health, WHO and through the MAAP consortium networks, in this order (S3 File supporting information).

Data on national Gross Domestic Product (2017 & 2018) and population data were collected from the World bank database [14]. Global Health AMR prevention index 2019 were collected from the Global Health Security Index data base 2019 [15], mortality to infectious conditions (2019) were collected from the WHO Health observatory [16] and number of outbreaks reported between 2016 and 2018 were extracted from the report of Talisuna and colleagues [17].

### Statistical analysis

Country-level AMC levels were derived from the various combinations of available data collected through the backfilling methodology. The country-level consumption of each drug was expressed as defined daily dose (DDD) and DDD per inhabitant (DID), calculated from data collected at national level. DDD is the assumed average maintenance dose per day for a drug used for its main indication in its target species [8]. The total antimicrobial consumption in milligrams per country per year was divided by its standard DDD value in milligrams documented by the WHO to obtain the total DDD per year per country. The DID represents the number of people in a population of 1,000 treated daily with a particular antimicrobial or group of antimicrobials. DID were calculated based on the national consumption and population figures. For each country, levels of antibiotic consumption at facility and national level were determined from aggregated data from the private and public sector, as applicable.

Antibiotic consumption patterns at national and facility level were examined through the relative proportions of AWaRE categories and through their level of compliance to the WHO global target of at least 60% of total consumption being Access group antibiotics. Percentage consumption of Access drugs at facility level were compared to national levels using the 95% confidence intervals around the mean. Association between achieving the threshold of 60% consumption of Access drugs and being a hospital or a community pharmacy was examined using the chi-square. Correlation between two sets of data (*e.g.,* DID and national GDP) was done using the Pearson correlation test. The spectrum of individual and groups of molecules most used at country and at facility level, was determined based on the drug utilization 75% indicator (DU75) [18] and the relative proportion of the Anatomical Therapeutic Chemical (ATC) categories.

### Ethical approval

The study did not involve human subjects and did not require ethical approval. Umbrella data sharing agreements were obtained from relevant health authorities and covered data collection and analysis across all participating agencies and facilities in each of the 14 countries. In addition, consent to share data was sought from importers, wholesalers, distributors and facilities, before starting data collection.

## Results

### Sources contributing AMC data

National data from public central medical stores were collected from nine of the 14 participating countries (Table 1), covering 100% of antibiotic trade through public channels. No data were collected from public central medical stores in Sierra Leone, Nigeria, Ghana and Zambia due to unavailability of central medical store at the time of data collection (Sierra Leone), political unrest (Burkina Faso), unusable data format (Zambia), unfeasibility of collection due to the high number of data sources (Nigeria counting 236 medical stores across the federal state), or access not granted by public authorities (Ghana). Country-level data from pools of private distributers and wholesalers were part of the MIDAS database for six of the 14 participating countries (Table 1), representing 40% to 100% of antibiotic trade through private channels: Uganda (40%), Kenya (85%), Cameroon (99%), Gabon (95.6%), Burkina Faso (99.5%) and Senegal (100%). Import data could be collected in one (Sierra Leone) of the four countries without any information available from private wholesalers and distributers nor public central medical stores. A total of 327 pharmacies contributed data to the study (Table 1). Data from both hospital and community pharmacies were collected in 12 of the 14 countries. The eligible community pharmacies of Cameroon and Uganda had inadequate system to track the sales of antibiotics, had no information covering the survey period or were unwilling to share their data.

**Table 1:** Sources providing AMC data to the study.

Countries	Sources of country-level AMC data providing data and associated coverage (in %)	Number of hospital pharmacies providing data	Number of community pharmacies providing data	Period of data collection
**Burkina Faso**	Wholesalers [IQVIA (99.5%)]	11	14	2017 - 2019
**Ghana**	**None**	11	15	2016 - 2019
**Cameroon**	Wholesalers [MIDAS (99%)], public central (100%) medical store	11	0	2017 - 2019
**Nigeria**	Wholesalers [MIDAS (100%)]	25	27	2016 - 2018
**Sierra Leone**	Import	7	7	2017 - 2018
**Malawi**	Public central medical store (100%)	14	7	2016 - 2018
**eSwatini**	Public central medical store (100%)	11	7	2016 - 2018
**Kenya**	Wholesalers [MIDAS, 85%)] public central medical store (100%)	15	10	2016 - 2018
**Uganda**	Wholesalers [MIDAS (40%)] public central medical store (100%)	11	0	2017 - 2018
**Senegal**	Wholesalers [MIDAS (100%)] public central medical stores (100%)	11	6	2017 - 2018
**Gabon**	Wholesalers [MIDAS (95.6%)] public central medical store (100%)	6	18	2016 - 2018
**Tanzania**	Public central medical store (100%)	16	7	2016 - 2018
**Zimbabwe**	Public central medical stores (100%)	13	19	2017 - 2018
**Zambia**	**None**	14	14	2016 - 2018

All data from central medical stores and from wholesalers providing information to MIDAS were available in electronic format (S1 Fig). Twenty-three percent of hospital pharmacies participating in the study had electronic data system, against 55% of community pharmacies. The year 2017 and 2018 were consistently represented among available data from all sources and across countries and served as index years for all subsequent analysis.

### Levels and patterns *of* country and facility-level antibiotic consumption

**Fig 3:**
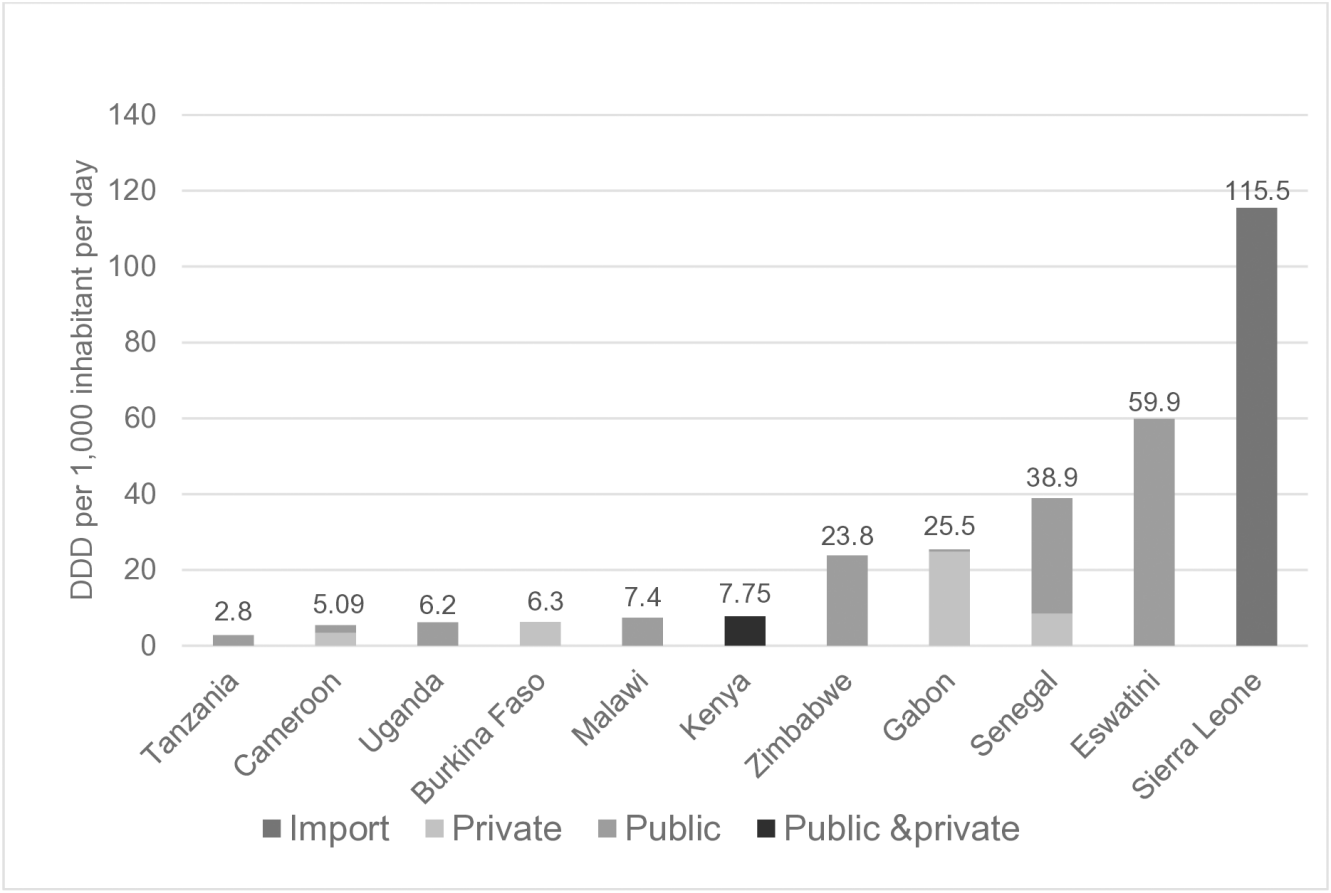
DDD per 1,000 inhabitants per day (DID) in available data sources from 11 participating countries.

**Fig 4:**
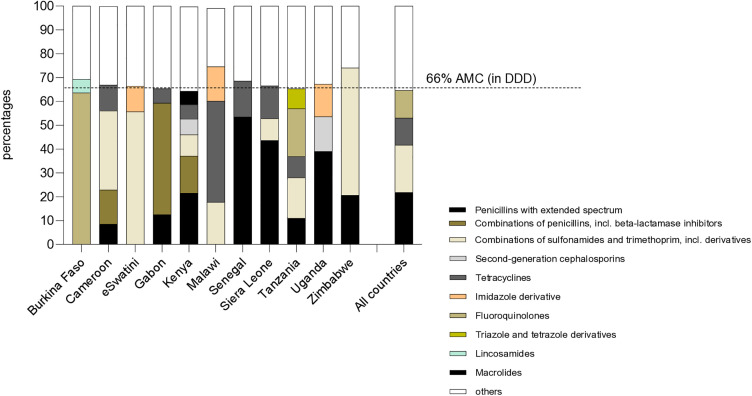
Proportion of ATC categories contribution to 66% AMC. Percentage of ten sub-categories of the JO1 ATC class of antibiotics relative to the total national AMC expressed in DDD. Only the largest sub-categories contributing 66% of the total AMC are shown. Sub-categories with smaller relative contributions to the total AMC are aggregated as ‘others’. Author analysis based on IQVIA private sector pharmaceutical sales data 24 months up to and including the month of December 2018, IQVIA Solutions (Pty)Ltd. All right reserved; S1 Appendix.

Comprehensive (i.e., ≥ 85% coverage) national-level data from all public, private for-profit and private not-for-profit markets were available from four of the 14 participating countries (Cameroon, Kenya, Gabon and Senegal, Table 1). Overall, 4.674 billion DDD were consumed by 555.2 million inhabitants across the 11 countries with data available at central level. Antibiotic consumption corrected for country population size was 8.42 for the pool of 11 countries, varying from 2.8 in Tanzania to 115.5 in Sierra Leone (Fig 3). Among the four countries with comprehensive AMC data across the public and the private sector, the consumption of antibiotic per inhabitant in Gabon and Senegal were five to seven time higher than the consumption per inhabitant in Cameroon and Kenya. AMC was largely driven by the private sector in Gabon (25 versus 0.7), by the public sector in Senegal (8.45 versus 30.5) and more equally distributed between the two sectors in Cameroon (3.4 versus 2.1). The means of 2017 and 2018 DID and percentage of DID change between 2017 and 2018 did not correlate with 2017 or 2018 GDP, aggregated mortality, malaria-, diarrhoea-, tuberculosis- and lower respiratory infection-associated mortality (2018) number of outbreaks reported between 2016 and 2018 or Global Health security AMR prevention index (2019) (S1 Table). Overall, 80% of the country-level AMC was related to five ATC classes [Penicillin with extended spectrum (34,48%), combination of sulphonamide and Trimethoprim (15.71%), Tetracyclines (13.12%), Fluroquinolones (10.13%) and combination of penicillin, including beta-lactamase inhibitors (6.22%), [Fig 4]. One ATC class represented more than half of the consumption in Burkina Faso (64,5% fluoroquinolones), eSWatini and Zimbabwe (55.7% and 53.4% of penicillin with extended spectrum, respectively).

**Fig 5:**
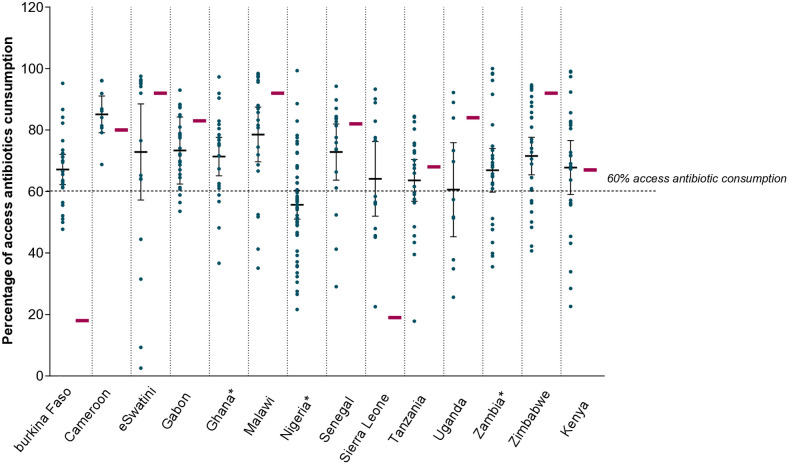
Percentage of Access antibiotic consumption at facilities and at national level: distribution per country. Percentages of Access drugs in AMC were calculated out of total antibiotic consumption. For each country, the distribution (mean, and 95% CI) of percentage Access drugs across participating facilities is shown alongside the percentage of Access drugs within the national consumption (in red). *No national AMC data were available in Ghana, Nigeria and Zambia. Values above the dotted lines are compliant with the WHO AWaRe target of at least 60% consumption from Access drugs. Author analysis based on IQVIA private sector pharmaceutical sales data 24 months up to and including the month of December 2018, IQVIA Solutions (Pty)Ltd. All right reserved; S2 Appendix.

In nine of 11 (82%) countries with data available at central level, drugs from the Access category of the AWaRe classification accounted for more than 60% of the total antibiotic consumption [**Fig 5**], in line with WHO recommendations. Mean of relative consumption of Access drugs was significantly lower in facilities as compared to national levels in eSwatini, Malawi, Uganda and Zimbabwe, significantly higher in facilities as compared to the national level in Burkina Faso and Sierra Leone, and comparable between facility and national level in Kenya, Tanzania, Gabon and Cameroon (Fig 4 proportion of ATC categories contribution to 66% AMC and S1 Appendix). The mean relative proportion of Access drugs in the total AMC in facilities was higher than 60% in all countries except in Nigeria (56%). In Zambia, community pharmacies were significantly more represented than hospital pharmacies among facilities not achieving the 60% consumption from of Access drugs (X2 = 4.762; p = 0.0291, S2 Table). Conversely, hospital pharmacies were significantly more represented than community pharmacies among the facilities not achieving the 60% consumption from Access drugs in eSwatini (X2 = 5.657; p = 0.0174). The representation of hospital and community pharmacies was otherwise comparable among facilities not achieving the 60% consumption of Access antibiotics in other countries. Overall, antibiotics from the Reserve category were procured by five of the 11 countries with national data available and accounted for 0.01% of the total national AMC. Reserve drugs were dispensed in 17 of the 327 (5%) participating pharmacies across five countries [eSwatini, (N = 6), Nigeria & Tanzania (N = 1), Uganda (N = 2) and Kenya (N = 7)].

### DU75 *in* participating facilities

**Fig 6:**
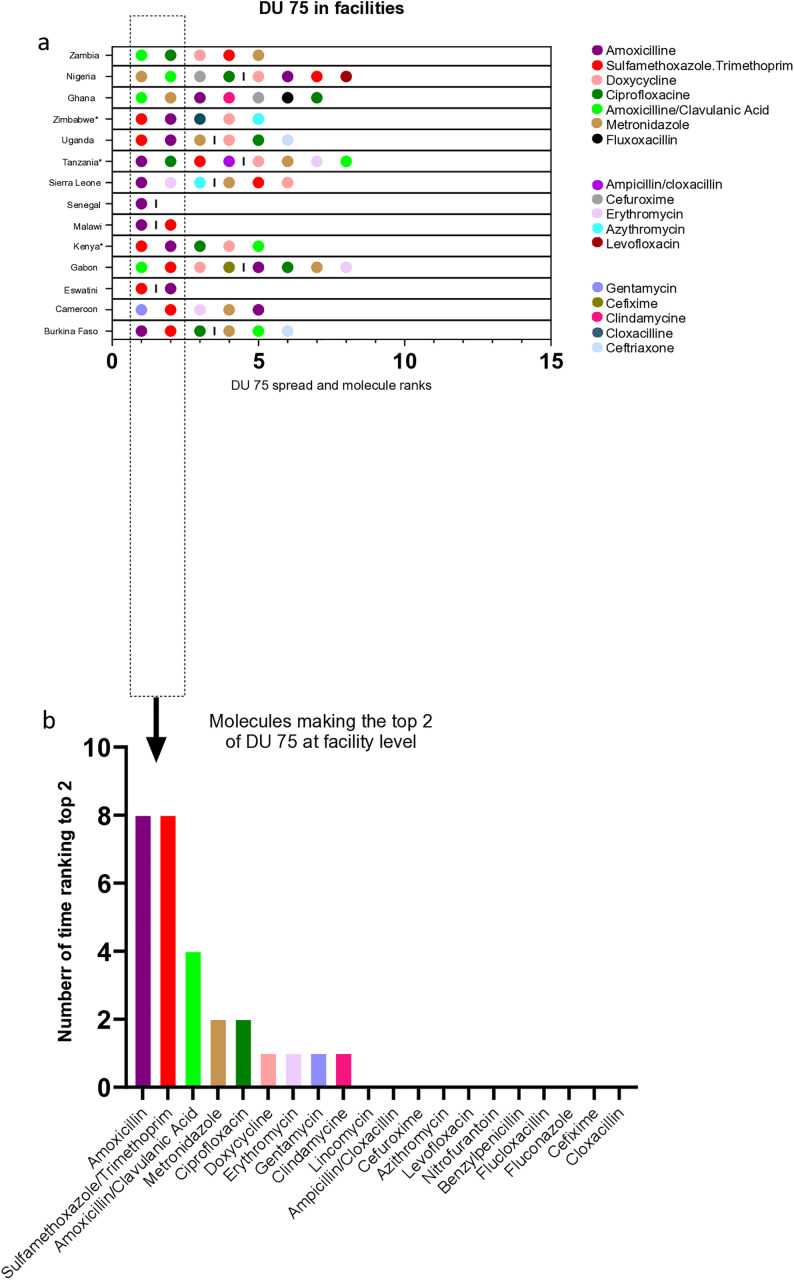
Spectrum and frequency of molecules constituting the DU75 at facility level. (a) Country drug utilization index 75% (DU75) is calculated from aggregated values reported by all participating facilities in individual countries. (b) the bars represent the number of countries where a given antibiotics makes the top 2 of the DU75.

A total of 17 molecules were represented at least once in the DU75 of facilities [**Fig 6**]. They included nine (53%) antibiotics from the Access category, seven drugs (41%) from the Watch category and one (6%) non-recommended drug combination (Ampicillin/cloxacillin). The mean number of individual molecules contributing to 75% of the total AMC was 4.85 at facility level (varying from one in Senegal to eight in Nigeria, Kenya and Gabon, Fig 6a: spectrum and frequency of molecules constituting the DU75 at facility level, DU75 in facilities and S3 Appendix). Amoxicillin and Sulfamethoxazole/Trimethoprim were most frequently represented in the top two of DU75 in eight of the 14 countries (Fig 6b: spectrum and frequency of molecules constituting the DU75 at facility level, molecules making the top two of DU 75 at facility level). This was followed by Amoxicillin/Clavulanic acid (4/14), Metronidazole and Ciprofloxacin (2/14) and Doxycycline, Erythromycin, Gentamycin and Clindamycin (1/14). Collectively, the Access drugs in facility DU75 represented only 10% of all possible Access drugs recommended by WHO within the J01 and J02 ATC categories (S2 Fig): one in 11 JO1A; zero of 3 JO1B; four of 25 J01C; zero of 11 JO1D; one in 33 JO1F; one in two; one in five JO1X; and zero of one J02A.

### Comparison of antimicrobial consumed with national and WHO essential Medicine lists

**Fig 7:**
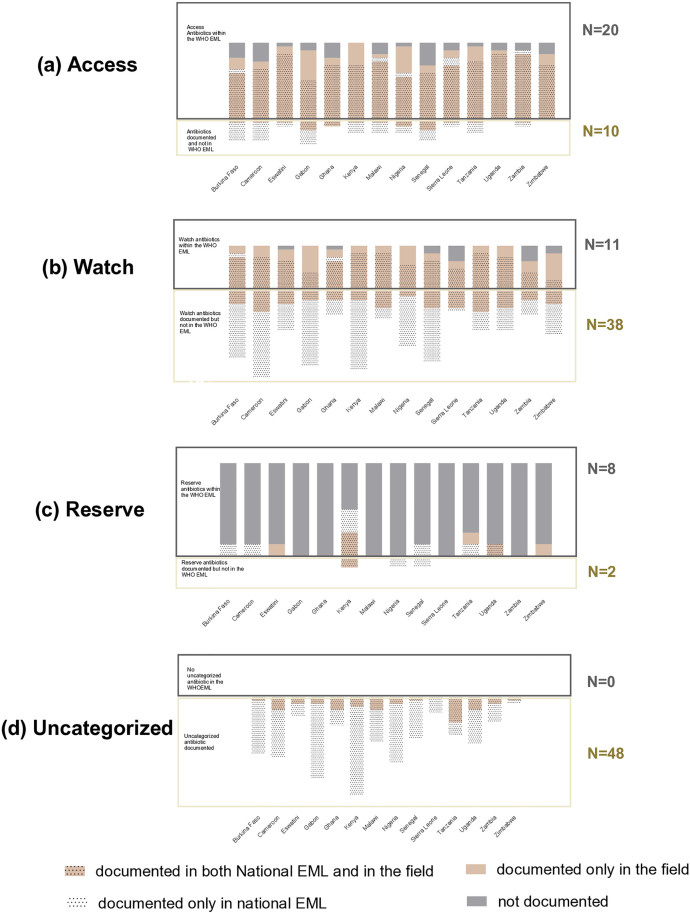
WHO categorized (Access, Watch and Reserve) and uncategorized antibiotics included in national EML or circulating in country. The top part of the graphs describes the situation of antibiotics listed in the WHO EML, i.e., (i) documented in the national EML; and/or (ii) documented in the field; or (iii) not documented. The bottom part of the graphs describes the situation of antibiotics not listed in the WHO EML, i.e., (i) documented in the national EML; and/or (ii) documented in the field.

National EML covered an average of 14 of the 20 (70%) Access drugs in the WHO EML ranging from ten (Gabon) to 18 drugs (Zambia). [**Fig 7a**]. An average of three WHO-recommended Access drugs that were not included in respective national EML were documented in the national or pharmacies’ files of 13 countries, ranging from one (Malawi and Uganda) to eight drugs (in Gabon). Twelve of the national EML included one (Burkina Faso, Cameroon, eSwatini, Malawi, Kenya, Sierra Leone, Tanzania, Zambia) to three (Gabon, Senegal) WHO non-recommended Access antibiotics. Additionally, one to five WHO non-recommended Access antibiotics were documented in 11 countries, despite not being included in the respective national EML.

National EML covered an average of seven (60%) of the 11 Watch drugs in the WHO EML, ranging from two (in Zimbabwe, Fig 7b) to nine (in Burkina Faso, Kenya, Malawi and Tanzania). An average of three WHO-recommended Watch drugs that were not included in respective national EML were documented in all 14 countries. WHO non-recommended Watch antibiotics not included in national EML were documented in all countries and reaching more than 13 drugs in six of the 14 countries.

Only six countries included antibiotics from WHO EML Reserve categories in their national EML [Burkina Faso, Cameroon, Senegal, Tanzania, Uganda (N = 1) and Kenya (N = 4), Fig 7c]. The WHO-recommended Reserve drugs in national EML were documented in two of the five countries (Uganda and Kenya). WHO-recommended Reserve antibiotics, not included in national EML were documented in eSwatini, Tanzania and Zimbabwe. The national EML of Kenya, Nigeria and Senegal each included one WHO non-recommended Reserve antibiotics. Of these, only one was documented to circulate in Kenya

A total of 48 different uncategorized drugs (including 28 fixed drug combinations) were documented in national EML and from national and pharmacies files, across the 14 countries (Fig 7d). The uncategorized drugs documented ranged from two in Zimbabwe, to 28 in Kenya.

## Discussion

This study was undertaken to investigate the rates, trends and patterns of AMC for human health in 14 African countries. The collective DID of 8.4 reported here is lower than the 2018 global (20.6) and regional modelled estimates [13.1 (95%, CI 11.8–14.6] previously reported [19] and shows large disparities between countries. But given the incomplete coverage of AMC data in seven of the 11 countries with national data available, the national DID estimates might reflect segmented intra-sector antibiotic trade, or reflect the exportability, availability and scope of data sources, rather than actual differences in antibiotic consumption. For instance, the import data collected in Sierra Leone might not capture possible subsequent export of antibiotics to other countries, thereby overestimating the Sierra Leonean Ciprofloxacin consumption. In the four countries with comprehensive datasets (Cameroon, Gabon, Senegal and Kenya) the disparities in consumption levels and relative size of private and public antibiotic trade, warrant additional investigations to understand how healthcare organization, market incentives, disease epidemiology or prescription practices might shape AMC in country. For instance, DID level was five-time higher in Gabon as compared to its neighbouring country Cameroon. The consumption attributed to the private sector was 97% in Gabon compared to 49% in Cameroon, in a context where the size of private healthcare services is comparable between the two countries (51% versus 53% in Cameroon) [20,21].

Generally, the current national AMC data systems allowed the determination of relative proportion of ATC classes and AWaRe categories consumed, but were unsuitable to comprehensively gauge volumes of consumption, help determine whether this consumption is larger in the private versus the public sector or compare AMC level between countries and over time. AMC data systems were also not suited to track the movement of antibiotics from central medical stores or wholesalers to other countries or the transactions between the private and the public sectors. Yet, simple tweaks in national data collection systems, like ‘distributed to’ or ‘public/private provider’ information, could support more granular sub-national and sector specific analysis.

The low percentage of electronic transaction systems in hospital pharmacies parallels our previous observation revealing that less than 15% of laboratories in the participating hospitals, use electronic-based laboratory data system11 [1] and that only 12% of AMR laboratory record include patient information [11]. Our findings further emphasize that the lack of interoperable electronic information systems supporting the collection and integration of patient history, clinical data and AST results prevents the determination of patient-level antimicrobial use, the routine analysis of concomitance between AMC, AMU and AMR and the implementation of evidence-based antibiotic stewardship [3,12].

Nine of the ten countries with available national AMC data achieved the WHO AWaRe target of 60% consumption from antibiotics in the Access category [7]. The very low percentage of Access antibiotic in the national AMC of Burkina Faso could reflect a private sector-driven consumption of Watch antibiotics. The low relative consumption of Reserve antibiotics observed in all participating countries corroborates previous estimate of 0.03% of Reserve use in the region [22]. The achievement the AWaRe target (60% consumption of Access drugs) and the low relative consumption of Reserve drugs do not by themselves exclude the possibility of over or under-use of Access antibiotics nor confirm a lack of access to Reserve antibiotics. Additional information on infectious disease burden, actual rates of AMR and baseline access to antibiotic are needed to evaluate appropriate antibiotic use. Collectively, these observations support the recommendations of Mendelson and colleagues [23], underscoring that despite the value of the WHO global standardized targets for antibiotic consumption, the identification of timely and locally relevant measures for appropriate antibiotic use, requires the adoption of additional national or local risk-adjusted targets for accessibility and use of certain antibiotics. Disease burden-adjusted use might for instance, be more relevant that total use indicators. Setting context-relevant and feasible indicators of AMC should be considered a priority by country AMRCC and an integral part of National AMR action plans.

Regardless the level of uncertainty around some DID estimates, our finding suggests that countries access but a narrow range of antibiotics. Five ATC sub-classes classes represented 80% of distributed or retailed antimicrobials. In addition, 75% of the consumption and dispensation was accounted for by an average of less than five antibiotics at facility level. Finally, most consumed or dispensed Access antibiotics, represented but a small selection of all possible WHO recommended Access drugs within individual ATC categories. The poor and delayed access to antibiotics has been documented in all regions of sub-Saharan Africa [3,24] and is recognized to cause more deaths than AMR [25,26]. The lack of information on therapeutic indication, prescription and administration of antibiotics in the participating facilities, hampers the ability to judge the suitability of antimicrobial use. Amoxicillin (alone or combined clavulanic acid) represents the most consumed antibiotic at facility level and is also the recommended first-line drug for nine of the ten most common mild community infections [7]. In contrast, the relatively large and narrow (*e.g.,* in Senegal) consumption of sulfamethoxazole/trimethoprim, could indicate a high prevalence of lower urinary tract infection and infectious diarrhea [7] or injudicious prescriptions. The frequent consumption ampicillin/cloxacillin reported in Tanzania, corroborates previous results [27] and suggests poor antimicrobial stewardship practices, given the lack of evidence for the clinical utility of this fixed drug combination. Additionally, the dispensation of Reserve antibiotics in only seven of the 64 tertiary hospitals participating in the study might indicate that appropriate and effective treatment are not available to hospitalized patients with multidrug-resistant bacteria. Finally, the concomitant analysis of AMR in participating hospitals published elsewhere [21] revealed high AMR prevalence in WHO critical and high priority pathogens. The reasons underlying the poor access to Reserve antibiotics in the participating countries might be related to insufficient bandwidth of national drug agencies to conduct the trials required for obtaining marketing authorization; the lack of scientific evidence available to physicians for routine clinical use [28]; the low affordability or the lack of diversity in drug formulation as described in other LMIC settings [29]. Regardless the root causes of their poor availability, providing access to Reserve antibiotics for patients who need them should be accompanied by robust mechanisms restricting and tracking the use of these drugs ensuring their prescription solely for the management of severe multi-resistant bacteria as recommended by the WHO [7]. This can be done through identifying novel ways to reinforce higher-level regulatory frameworks around prescription practices [30] and increase the effectiveness of antibiotic stewardship programmes at all levels of the health care system. Additionally, National Health Authorities could make the reporting of Reserve antibiotic sales by pharmaceutical companies or retailers mandatory, as currently done for all antibiotics sold in the veterinary sector of the European Union [31] and the United Kingdom [32].

In general, national EMLs included several WHO classified non-recommended and WHO unclassified molecules. Regardless the composition of national EMLs, the full range of WHO-recommended Watch molecules was documented in eight of the 14 countries. In contrast, the full range of WHO-recommended Access molecules was documented only in Kenya, and the full range of WHO-recommended Reserve molecules was not documented anywhere. The consumption of Watch molecules and fix drug combinations is reportedly on the rise in LMIC [22,27,33]. Given the context of limited AMR diagnostic options and ineffective AMR surveillance systems [11,21] in the participating countries, the relatively higher availability of diverse Watch molecules and fixed drug combinations suggested here and reported elsewhere [23], carry the risk of fuelling the emergence of AMR.

The fact that national EML do not cover the full range of AWaRe molecules is not necessarily indicative of inappropriate policy. It could reflect various scopes of local disease burden or country prioritization in the context of limited resources. However, the high number of circulating WHO non-recommended and unclassified drugs inside and outside national EML suggests a lack of knowledge of policy makers regarding the drivers of AMR, possible pressure from the local market to list unclassified drugs in national EML or to procure non-listed drugs and a lack of country capacity to control the circulation of unregulated drugs.

The main limitation of this study is the fragmented availability of the national-level data, which limits the comparability of AMC over time and across countries. Furthermore, the differences in prescription practices, population characteristics, disease burden or health policy approaches prevent drawing meaningful comparison across countries and warrants a cautious interpretation of the data at regional level. The selection of participating community pharmacies was skewed towards those agreeing to participate and those maintaining sufficient antibiotic transaction records. Moreover, the retrospective data collection strategy restricted our capacity to identify and correct data gaps, under-reporting or other data quality issues, which might have affected the outcome of the analysis. The result reported here represent the situation of AMC around hospitals initially selected for hosting laboratories capable of conducting AST. Therefore, extrapolation of the data to settings with less access to AST, might misestimate the judicious use of antibiotics.

Some recommendations emerge from our findings. Firstly, technology, regulatory and policy interventions aiming to increase the volume of analysable data on AMU, AMC and AMR merit a higher position among the priorities of the NAPs in LIMCs. These initiatives require intersectoral, multi-disciplinary and inter-ministerial collaborations and partnerships reaching far beyond the remit of AMR stakeholders and the scope of the One Health approach. Improvement of data systems could be conducted in the context of enforcing regulation for trade, safety of medicines and tax management of health products. The European Surveillance of Antimicrobial Consumption Network (ESAC-Net) provides a useful example of a multi-country framework for the collection, analysis and sharing of micro- and macro-level AMC data under the coordination of the European Centers for Disease Control and the umbrella of the European Union [34]. In the United Kingdom the Fingertip platform of the Department of Health & Social Care is a country-level tool for the systematic collection of AMC by the specialized unit of the National Health Agency [35]. Other opportunities to increase the availability of AMC data in high income countries include the mandatory reporting of antibiotic sales by pharmaceutical companies or tapping into existing data system for medical prescription or health care reimbursements from medical insurances. The continuous training of pharmacists as part of the systematic AMC data collection from hospital central medical stores and pharmacies coordinated by the Indian NAC-Net surveillance [36] offers a useful practical example of fit-for-context AMC surveillance strategies. Similarly to Uganda [37], LMICs have the opportunity to direct international aid such as the USAID or Global Fund and build the internal capacity of the National Drug Authority mandated to control import and export of medicines.

Secondly, the narrow scope of antibiotics consumption reported here underscores the need to accelerate equitable and sustainable access to antibiotics as part of strategies to achieve Universal Health Coverage and to combat AMR. Procurement and market shaping support similar to these developed for the deployment of drugs and diagnostic for the three big diseases (HIV, tuberculosis and Malaria) [38] could be applied to the accelerate access to antibiotics, as previously recommended by Laxminarayan and colleagues [26]. The COVID-19 pandemic has contributed to prioritizing local manufacturing as a key strategy to address the shortage of medical products while improving Africa’s self-reliance for Health Security and resilience [39]. National and regional policy makers and leaders should intensify the implementation of production-facilitating measures such as enabling policies, adequate regulatory frameworks, market shaping instruments (such as preferential procurement), tax breaks, ambitious research agendas and innovative partnerships, which could ease the scale-up of competitive local manufacturing of high-quality, affordable antibiotics on the continent [40]. Furthermore, the harmonization of EML and their alignment to the WHO recommendations should be considered by policy makers as matters of the greatest importance and priority. Countries and regions have an opportunity to tap into the expertise available at WHO country offices and in Regional Coordinating Centers of the Africa Centers for Disease control to improve their policy documents and keep them updated. Strategies and incentives contributing to the evidence-based selection of antibiotics in national EML and facilitating the enforcement of those lists are needed. Additionally, countries need to factor locally relevant estimates of disease burden when monitoring the judicious use of antibiotics.

## Conclusion

Despite the limited availability of AMC data, our findings highlight access to a poorly diversified subset of antibiotics. In the context of decreasing global health investments, governments should expand domestic financing and leverage existing global health funding to establish robust digital health technologies in support of stronger stewardship practices. Addressing supply chain barriers and ensuring equitable access to essential medicines are highlighted as critical approaches to curb the emergence of AMR.

## Supporting information

S1 File Supporting information: Fleming fund criteria for country prioritization. (DOCX)

S2 File Supporting information: MAAP collector and MAAP store applications. (DOCX)

S3 File Supporting information: Reference of National essential medicine lists. (DOCX)

S1 Table Correlation between the (A) mean 2017–2018 DID, the (B) percentage DID change between 2017 and 2018 and other economic and health variables. (DOCX)

S2 Table Proportion of hospital versus community pharmacies among facilities not achieving the 60% access drugs AMC. (DOCX)

S1 Fig Distribution of data format per type of AMC data source. (DOCX)

S2 Fig Percentage of access drugs represented in DU75 among all available access drugs regulated by WHO and disaggregated by ATC classes. (DOCX)

S1 Appendix Data for figure 4. (XLSX)

S2 Appendix Data for figure 5. (XLSX)

S3 Appendix Data for figure 6. (XLSX)
