# Supplementary material for: Antibiotic consumption in 14 countries of sub-Saharan Africa: Findings from a retrospective analysis
Source: PLoS One. 2025 Oct 30;20(10):e0333842. doi: 10.1371/journal.pone.0333842 (PMC12574848; doi:10.1371/journal.pone.0333842)
Supplement: S1 File — (DOCX) [file pone.0333842.s001.docx]

**S1: Supporting information**: Fleming Fund criteria for country prioritization

The Fleming Fund country selection was undertaken by applying a set of requirements from the funder – the UK Dept of Health and Social Care. A mixture of approaches were used based on different parameters, assessed through consultations with stakeholders and desk-based assessments.

The starting point was the list of all Overseas Development Aid (ODA) countries (n=146) to which we applied criteria for exclusion, aligned with the requirements of DHSC. Countries outside the Area of Operations were excluded – countries outside of sub-Saharan Africa, South Asia, and South-East Asia. Also, we excluded countries with income levels that were too high (upper-middle and middle-middle income), those with relevant active conflict, and those countries subject to UK or other sanctions. This step removed 99 countries.

Of the remaining 47 countries, 32 were selected based on being already selected as Early Investment Countries (EICs) (n=5); receiving grants from the Fleming Fund through the tripartite group (WHO, FAO and OIE) for developing National Action Plans; and other variables. The initial draft list resulting from that process produced an initial list of 33 countries.

Desk-based assessments were then undertaken in the 33 selected countries. Criteria used to assess countries through desk-based assessments were as follows:

- Assessment of progress towards establishing a surveillance system.
- Political will to tackle AMR
- Opportunity for Fleming Fund Investment(s)
- Additional criteria were then factored in: countries being hotspots for the interface between poverty, emerging livestock systems and zoonoses, countries with high levels of visitor exchanges with the UK, countries with clinical syndromes associated with bacterial infections as leading causes of ill health in 2015, being a UK Aid priority country, countries receptive to Fleming Fund funding.

The desk-based assessment produced a final list of 24 Fleming Fund priority countries and 9 reserve countries.
