# Supplementary material for: Antibiotic consumption in 14 countries of sub-Saharan Africa: Findings from a retrospective analysis
Source: PLoS One. 2025 Oct 30;20(10):e0333842. doi: 10.1371/journal.pone.0333842 (PMC12574848; doi:10.1371/journal.pone.0333842)
Supplement: S2 File — (DOCX) [file pone.0333842.s002.docx]

**S2: supporting information:** MAAP Collector and MAAP Store applications

The MAAP collector is a data collection application, specifically designed to collect antibiotic consumption, patient visits and lab studies over different sites.

The tool can be used on-site (in which case, connectivity is not the best) or with other systems that gather the information and have exporting capabilities, it can work offline and automatically synchronizes the data when connection is available.

The app supports:

- Importing of lab records and pharmacy stock information via .csv or .xls files
- Manual upload of patient information or pharmacy stock movements
- Obfuscation of sensitive patient information (also known as PHI)
- The application's installers are built for Windows and MacOS, and were built to get a good performance on devices with limited resources.

There’s one collector instance per country and it synchronizes with a its correspondent MAAP store.

This way the country decides where and how they want to deploy the server independently and guarantees full control over the access to the data.

The MAAP store application is the counterpart of the collector.

It covers a series of administrative tasks:

- Create users
  - Collectors (user of the collector app, no access to the server)
  - Viewers (access to the server with read capabilities)
  - Admins (access to the server with full capabilities)
- Create/Edit/Delete antibiotics, there's a pre-loaded list of antibiotics to simplfiy the data entry process for the collectors

It also offers access to all the data collected organized by area:

- Antibiotic consumption
- Electronic pharmacy stock records
- Laboratory records
- Patient information

The apps are Open source; there's more information in the repositories.

[MAAP Collector](https://github.com/instedd/maap-collector)

[MAAP Store](https://github.com/instedd/maap-store)
