# Supplementary material for: Antibiotic consumption in 14 countries of sub-Saharan Africa: Findings from a retrospective analysis
Source: PLoS One. 2025 Oct 30;20(10):e0333842. doi: 10.1371/journal.pone.0333842 (PMC12574848; doi:10.1371/journal.pone.0333842)
Supplement: S3 File — (DOCX) [file pone.0333842.s003.docx]

**S3 supporting information:** Reference of National Essential Medicine Lists

1. EML Kenya - Republic of Kenya, Ministry of Health. (2019). Kenya Essential Medicines List - 2019. Retrieved January 21, 2021, from https:// [www.health.go.ke/wp-content/uploads/2020/03/Kenya-Essential-Medicines-List-2019.pdf](http://www.health.go.ke/wp-content/uploads/2020/03/Kenya-Essential-Medicines-List-2019.pdf)
2. EML Burkina Faso - Ministere de la Sante. (2020). Liste Nationale des Medicaments Essentiels et Autres Produits de Sante . Ministere de la Sante.
3. EML Zimbabwe - Ministry of Health and Child Welfare, Republic of Zimbabwe. (2015). EDLIZ 2015: 5th Essential Drugs List and standard treatment guidelines for Zimbabwe. Harare: The National Drug and Therapeutics Policy Advisory Committee [NDTPAC].
4. EML Cameroon - Republique du Cameroun. (2017). Liste Nationale des Medicaments et Autres Produits Pharmaceutiques Essentiels. Republique du Cameroun.
5. EML Ghana - Ministry of Health; Republic of Ghana, 2017. Ghana National Drugs Programme: Essential Medicines List; Seventh Edition (7th). [Online] Available at: <https://www.moh.gov.gh/wp-content/uploads/2020/07/GHANA-EML-2017.pdf> [Accessed 2 September 2021].
6. EML Gabon - LISTE NATIONALE DES MEDICAMENTS ET DISPOSITIFS MEDICAUX ESSENTIELS EDITION 2019. Available at <https://cdn.who.int/media/docs/default-source/essential-medicines/national-essential-medicines-lists-(neml)/afro_neml/gabon-2019.pdf?sfvrsn=2b66899c_3&download=true>
7. EML Eswatini - Eswatini Essential Medicines List. (2012). Standard Treatment Guidelines and Essential Medicines List of Common Medical Conditions in the Kingdom of Swaziland. Retrieved from <https://www.medbox.org/pdf/5e148832db60a2044c2d2d3e>
8. EML Malawi - Malawi Standard Treatment Guidelines (MSTG) incorporating MEML. (2015). Malawi Standard Treatment Guidelines (MSTG) 5^th^ Edition. Retrieved from <https://extranet.who.int/ncdccs/Data/MWI_D1_Malawi-Standard-Treatment-Guidelines-Essential-Medicines>-List-2015.pdf
9. EML Nigeria - Federal ministry of health Abuja, Nigeria; Nigeria Essential Medicines List - 6th Edition 2016
10. EML Sierra Leone - Ministry of Health and Sanitation, 2016. National Essential Medicines List for Sierra Leone 2016, Freetown: Government of Sierra Leone.
11. EML Senegal - Senegal Essential Medicines List. (2018). Republic of Senegal. Retrieved from <https://www.who.int/medicines/areas/>coordination/senegal_eml.pdf
12. EML Zambia - Government of the Republic of Zambia, 2013. Zambia Essential Medicines List (ZEML), s.l.: Government of the Republic of Zambia
13. EML Tanzania - United Republic of Tanzania, Ministry of Health. (2018). Standard Treatment Guidelines and National Essential Medicines List Tanzania Mainland. Retrieved from <http://www.tzdpg.or.tz/fileadmin/documents/dpg_internal/dpg_working_groups_clusters/cluster2/health/Key_Sector_Documents/Tanzania_Key_Health_Documents/STANDARD_TREATMENT_GUIDELINES__CORRECT_FINAL_USE_THIS-1.pdf>
14. EML Uganda - Ministry of Health Republic of Uganda, 2016. Essential Medicines and Health Supplies List for Uganda (EMHSLU)2016., Kampala: Ministry of Health.
