# Supplementary material for: Antibiotic consumption in 14 countries of sub-Saharan Africa: Findings from a retrospective analysis
Source: PLoS One. 2025 Oct 30;20(10):e0333842. doi: 10.1371/journal.pone.0333842 (PMC12574848; doi:10.1371/journal.pone.0333842)
Supplement: S1 Table — (DOCX) [file pone.0333842.s004.docx]

**S1 table** correlation between the (A) mean 2017-2018 DID, the (B) percentage DID change between 2017 and 2018 and other economic and health variables

|  | 1. Mean DID 2017-2018 | | (B) % DID change between 2017 and 2018 | |
| --- | --- | --- | --- | --- |
|  |  |  |  |  |
| Variables | Correlation (spearman) | p (2-tailed) | Correlation (spearman) | p (2-tailed) |
| GDP 2017 | 0.2364 | 0.2364 | -0.3939 | 0.2632 |
| GDP 2018 | 0.3455 | 0.3304 | -0.3455 | 0.3304 |
| GDP change 2017-2018 |  |  | -0.2242 | 0.5367 |
| GHS AMR prevention index | 0.1646 | 0.6483 | -0.01231 | 0.98 |
| Life expectancy | -0.3091 | 0.3869 | -0.4438 | 0.1992 |
| number of outbreaks reported between 2016 and 2018 | -0.1596 | 0.7077 | -0.386 | 0.094 |
| Aggregated mortality (females) | 0.0303 | 0.946 | 0.1394 | 0.702 |
| Aggregated mortality (males) | 0.1758 | 0.6321 | 0.1636 | 0.6567 |
| Mortality associated with LRI | -0.05455 | 0.8916 | 0.4061 | 0.2475 |
| Mortality associated with malaria | -0.5714 | 0.1511 | 0.3333 | 0.4279 |
| Mortality associated with tuberculosis | 1 | 0.0833 | -0.2 | 0.9167 |
| Mortality associated with diarrhea | -0.1423 | 0.717 | 0.3096 | 0.4155 |
| Total mortality of the top 10 infectious diseases | 0.1758 | 0.6321 | 0.1636 | 0.6567 |
