# Supplementary material for: Antibiotic consumption in 14 countries of sub-Saharan Africa: Findings from a retrospective analysis
Source: PLoS One. 2025 Oct 30;20(10):e0333842. doi: 10.1371/journal.pone.0333842 (PMC12574848; doi:10.1371/journal.pone.0333842)
Supplement: S2 Table — (DOCX) [file pone.0333842.s005.docx]

**S2 Table**: proportion of hospital versus community pharmacies among facilities not achieving the 60% access drugs AMC

|  | Hospital pharmacies | | Community pharmacies | | X^2^ | p |
| --- | --- | --- | --- | --- | --- | --- |
| Countries | ≥60% Access drugs | <60% Access drugs | ≥60% Access drugs | <60% Access drugs |  |  |
| Burkina Faso | 7 | 4 | 12 | 2 | 1.646 | 0.1995 |
| Cameroon | 10 | 0 | 0 | 0 | na | na |
| eSwatini | 11 | 0 | 4 | 3 | 5.657 | 0.0174* |
| Gabon | 5 | 1 | 17 | 2 | 0.163 | 0.6864 |
| Ghana | 9 | 1 | 9 | 4 | 1.433 | 0.2313 |
| Kenya | 12 | 4 | 5 | 4 | 1.001 | 0.3717 |
| Malawi | 12 | 2 | 5 | 2 | 0.617 | 0.432 |
| Nigeria | 8 | 16 | 10 | 17 | 0.076 | 0.7827 |
| Senegal | 9 | 2 | 5 | 1 | 0.006 | 0.9383 |
| Sierra Leone | 4 | 3 | 1 | 6 | 2.8 | 0.0943 |
| Tanzania | 10 | 7 | 5 | 2 | 0.336 | 0.5621 |
| Uganda | 6 | 5 | 0 | 0 | na | na |
| Zambia | 8 | 6 | 13 | 1 | 4.762 | 0.0291* |
| Zimbabwe | 10 | 2 | 12 | 6 | 1.023 | 0.3118 |
| **Total** | **121** | **53** | **98** | **50** |  |  |
