# Supplementary material for: Antibiotic consumption in 14 countries of sub-Saharan Africa: Findings from a retrospective analysis
Source: PLoS One. 2025 Oct 30;20(10):e0333842. doi: 10.1371/journal.pone.0333842 (PMC12574848; doi:10.1371/journal.pone.0333842)
Supplement: S1 Fig — (DOCX) [file pone.0333842.s006.docx]

**S1 Fig** Distribution of data format per type of AMC data source
