# Supplementary material for: Antibiotic consumption in 14 countries of sub-Saharan Africa: Findings from a retrospective analysis
Source: PLoS One. 2025 Oct 30;20(10):e0333842. doi: 10.1371/journal.pone.0333842 (PMC12574848; doi:10.1371/journal.pone.0333842)
Supplement: S2 Fig — (DOCX) [file pone.0333842.s007.docx]

**S2 Fig:** Percentage of access drugs represented in DU75 among all available access drugs regulated by WHO and disaggregated by ATC classes

Legend: The total number of Access drugs within each ATC sub-class served as denominator for the calculation of percentages. For instance, the J01A sub-class includes 17 Access drugs, of which 11 (65%) are recommended by the WHO. Only one of these 11 WHO-recommended drug (6%) was included in the pool of molecules represented at least once in country DU75.
